# FreeSurfer subcortical normative data

**DOI:** 10.1016/j.dib.2016.10.001

**Published:** 2016-10-14

**Authors:** Olivier Potvin, Abderazzak Mouiha, Louis Dieumegarde, Simon Duchesne, for the Alzheimer׳s Disease Neuroimaging Initiative

**Affiliations:** a Centre de recherche de l׳Institut universitaire en santé mentale de Québec, 2601, de la Canardière, Québec, Canada G1J 2G3; b Département de radiologie, Université Laval, 1050, avenue de la Médecine, Québec, Canada G1V 046

**Keywords:** Neuroimaging, Age, Sex, Magnetic resonance, Normality, Normal aging, Morphometry

## Abstract

This article contains a spreadsheet computing estimates of the expected subcortical regional volumes of an individual based on its characteristics and the scanner characteristics, in addition to supplementary results related to the article “Normative data for subcortical regional volumes over the lifetime of the adult human brain” (O. Potvin, A. Mouiha, L. Dieumegarde, S. Duchesne, 2016) [1] on normative data for subcortical volumes. Data used to produce normative values was obtained by anatomical magnetic resonance imaging from 2790 healthy individuals aged 18–94 years using 23 samples provided by 21 independent research groups. The segmentation was conducted using *FreeSurfer*. The spreadsheet includes formulas in order to compute for a new individual, significance test for volume abnormality, effect size and estimated percentage of the normative population with a smaller volume while taking into account age, sex, estimated intracranial volume (eTIV), and scanner characteristics. Detailed R-squares of each predictor for all formula are also reported as well as the difference of subcortical volumes segmented by *FreeSurfer* on two different computer hardware setups.

**Specifications Table**

**Table:** 

Subject area	*Neuroscience, Neurology, Neurobiology*
More specific subject area	*Volumetric subcortical normative values*
Type of data	*Tables, Excel file*
How data was acquired	*MRI images from open databases, data analyses and normative values generated by statistical models*
Data format	*Analyzed*
Experimental factors	*The sociodemographics, the scanner manufacturer and magnetic field strength*
Experimental features	*Subcortical volumes extracted using FreeSurfer*
Data source location	*Australia, Austria, Belgium, Canada, Finland, Germany, Ireland, Italy, Netherlands, United Kingdom, and USA*
Data accessibility	*Data is with this article*

**Value of the data**

• The data provides the first subcortical regional normative values in a very large sample of healthy individuals with a wide age range and diversity of scanner manufacturer and magnetic field strength.
• The calculator can be used to assess deviation from normality for any given individual patient or healthy control.
• These values can be useful for multicenter studies using various scanner manufacturers and magnetic field strengths.

## 1 Data

A Microsoft Excel spreadsheet computing expected subcortical regional volumes for an individual according to his age, sex, intracranial volume and the scanner characteristics is provided (see Subcortical_Norms_Calculator.xlsm file online). Table 1 reports detailed R-squares of each predictor for all models predicting subcortical volumes. Table 2 shows the difference of subcortical volumes segmented by *FreeSurfer* on two different computer hardware setups.

## 2 Experimental design, materials and methods

### 2.1 Participants and segmentation

A detailed description of the participants and segmentation procedure can be found in Potvin et al. [1].

### 2.2 Statistical analyses

Regression models predicting subcortical regional volumes were built using age, sex, eTIV, MFS, and scanner manufacturer as predictors. The details about model building can be found in Potvin et al. [1]. Individual predictors׳ weight was measured by squared semi-partial correlations.

The impact of the hardware setup on the volumes generated by *FreeSurfer* was tested by dependent one-sample *t*-tests with Bonferroni correction.

Detailed information about the normative statistics included in the Excel spreadsheet can be found in Potvin et al. [1] and in the work of Crawford and colleagues [2], [3].

## Figures and Tables

**Table 1:** Percentage of the variance explained (*R*^2^) by each predictor in models predicting subcortical regional volumes.

**Regions**	**Age**	**Age**^**2**^	**Age**^**3**^	**Sex**	**eTIV**	**eTIV**^**2**^	**eTIV**^**3**^	**MFS**	**GE / Siemens**	**Philips / Siemens**	**GE X MFS**	**Philips X MFS**	**eTIV X MFS**	**Age X Sex**	**eTIV X GE**	**eTIV X Philips**	**Total *R***^**2**^	**Validation *R***^**2**^
Accumbens L	25.6	1.1	–	1.6	0.2	0.0	–	1.0	0.4	1.4	1.8	5.1	–	0.4	–	–	38.5	34.2
Accumbens R	28.7	0.3	0.1	2.6	0.1	–	–	1.0	0.1	1.4	0.5	3.0	–	0.2	–	–	37.8	28.6
Amygdala L	14.0	1.1	0.1	13.1	4.2	0.1	–	8.1	0.0	0.0	0.5	0.0	–	0.1	0.1	0.1	41.4	39.0
Amygdala R	9.6	0.1	0.2	12.7	3.5	–	–	4.4	0.1	0.0	0.0	0.4	–	–	–	–	31.1	33.9
Brainstem	3.1	0.9	0.3	21.5	26.7	0.2	–	0.0	0.3	0.0	0.9	0.1	–	0.2	–	–	54.1	61.1
Caudate L	12.8	3.7	0.1	7.1	15.1	0.2	0.0	–	0.0	2.0	–	–	–	0.2	0.0	0.1	41.2	37.0
Caudate R	9.0	7.2	–	6.8	11.7	0.0	–	0.0	0.4	5.5	0.0	0.5	0.0	0.2	0.0	0.2	41.7	31.4
Hippocampus L	21.4	5.8	0.0	6.9	10.6	0.2	–	3.3	0.7	1.6	0.2	0.0	0.0	0.2	–	–	50.9	48.2
Hippocampus R	18.0	6.7	0.1	7.2	11.1	–	–	5.3	0.4	0.6	0.2	0.1	–	0.2	–	–	49.7	51.6
Pallidum L	14.5	3.0	0.1	8.8	8.6	0.2	–	1.4	0.6	0.6	0.0	1.8	–	0.6	–	–	40.0	37.8
Pallidum R	19.5	1.5	0.5	8.5	6.9	0.1	–	1.1	0.1	3.6	0.3	1.1	–	0.3	–	–	43.4	42.4
Putamen L	34.6	1.9	–	6.2	3.3	0.0	0.0	0.1	0.1	3.8	0.1	1.5	0.2	0.3	–	–	52.0	41.9
Putamen R	34.7	2.9	0.0	7.3	3.2	0.0	–	0.2	0.1	3.4	0.0	2.1	–	0.5	–	–	54.2	47.2
Thalamus L	27.3	1.8	0.4	10.8	17.1	0.5	–	1.7	0.0	0.8	0.1	0.5	0.2	0.3	–	–	61.5	57.3
Thalamus R	34.8	0.4	0.3	12.1	17.0	0.3	–	0.6	0.0	0.5	0.0	0.1	–	0.3	0.0	0.2	66.6	66.3
Ventral DC L	17.9	0.6	0.6	17.9	20.5	0.4	–	1.3	0.0	1.3	0.1	0.0	–	0.1	–	–	60.8	66.9
Ventral DC R	26.2	0.2	–	16.7	17.9	0.3	–	0.6	0.0	0.6	0.1	0.0	–	0.2	0.1	0.0	62.8	64.1
Ventricles^1^	40.2	3.3	–	4.9	7.6	–	–	0.0	0.1	0.4	–	–	0.0	0.3	0.0	0.1	56.9	66.9
Lateral L^1^	39.3	2.3	–	3.6	7.4	–	–	–	0.1	0.5	–	–	–	0.3	0.0	0.0	53.4	61.7
Lateral R^1^	38.6	2.8	–	4.0	6.9	–	–	–	0.1	0.3	–	–	–	0.2	0.0	0.1	53.0	65.2
Inferior lateral L^1^	21.7	9.0	0.0	4.4	1.0	0.2	0.0	1.5	0.1	1.1	0.7	0.0	–	0.3	0.1	0.2	40.4	43.4
Inferior lateral R^1^	16.0	8.9	0.4	3.3	0.2	–	–	1.9	0.0	1.4	–	–	0.2	0.5	0.0	0.2	33.0	32.6
3rd^1^	42.6	3.5	0.0	7.5	5.3	0.1	–	0.1	0.2	0.1	0.2	0.1	0.1	0.2	–	–	59.9	64.1
4th	0.2	0.7	–	6.4	5.1	0.0	0.1	0.8	0.5	0.0	–	–	–	–	0.1	0.1	13.9	11.4
Corpus callosum	17.7	5.0	0.2	2.0	6.5	0.0	0.1	2.4	0.2	0.0	–	–	0.1	–	0.4	0.1	34.8	32.7
Subcortical GM	41.0	0.1	0.0	15.5	16.8	0.2	–	0.8	0.0	0.5	0.1	0.3	–	0.4	–	–	75.6	72.0

^1^Log_10_ transformed. MFS: Magnetic field strength, eTIV: Estimated total intracranial volume. GM: gray matter.

**Table 2:** Subcortical volumes differences between segmentation on two different computer hardware setups (*n*=50).

**Regions**	**Mean difference (%)**	***t***	***p***
Accumbens L	0.05	−0.22	0.825
Accumbens R	1.10	0.97	0.339
Amygdala L	0.95	1.71	0.094
Amygdala R	0.96	1.79	0.080
Brainstem	0.09	0.6	0.552
Caudate L	0.02	−0.05	0.961
Caudate R	0.07	0.18	0.854
Hippocampus L	0.41	1.48	0.144
Hippocampus R	−0.55	−2.01	0.049
Pallidum L	−0.35	−0.46	0.645
Pallidum R	−0.37	−0.73	0.471
Putamen L	0.52	1.3	0.200
Putamen R	0.18	0.65	0.519
Thalamus L	−0.03	−0.17	0.862
Thalamus R	−0.11	−0.44	0.658
Ventral DC L	−0.07	−0.19	0.851
Ventral DC R	0.11	0.36	0.723
Ventricles			
All	0.00	−0.18	0.858
Lateral L	−0.01	−0.22	0.830
Lateral R	0.00	0.16	0.874
Inferior lateral L	−0.20	0.65	0.521
Inferior lateral R	0.15	−0.02	0.984
3rd	−0.06	−0.74	0.461
4th	−0.09	−0.09	0.928
Corpus callosum	0.34	0.91	0.366
Subcortical GM	0.10	0.90	0.375

Bonferroni-corrected critical value for significance: .002.
