# Corrigendum to: “FreeSurfer subcortical normative data” [Data in Brief 9 (2016) 732–736]

**DOI:** 10.1016/j.dib.2019.103704

**Published:** 2019-02-26

**Authors:** Olivier Potvin, Abderazzak Mouiha, Louis Dieumegarde, Simon Duchesne

**Affiliations:** a CERVO Brain Research Centre, 2601, de la Canardière, Québec, Canada G1J 2G3; b Département de radiologie, Faculté de médecine, Université Laval, 1050, avenue de la Médecine, Québec, Canada G1V 0A6

We have recently uncovered a flaw in our statistical method that impacts the semi-partial R^2^ results presented in Figure 1. These values were computed using the option *Effect size* within the *GLM* procedure of the SAS statistical software and are labeled by SAS as semi-partial eta squares. These values are, in fact, a partitioned R^2^ according to a given order of predictors׳ entry. The main impact of this procedure is that the order of the predictors influences the semi-partial R^2^. While it does not impact the regression models, for those readers that are drawing conclusions based on the relative importance of the predictors, we felt compelled to provide more accurate and robust results.

In all of our analyses, the predictors were listed in a given, unvarying order as presented in the article tables (age, age^2^, age^3^, sex, estimated intracranial volume (eTIV), eTIV^2^, eTIV^3^, magnetic field strength, GE manufacturer, and Philips manufacturer, followed by interactions). Therefore, the variables listed earlier were favored in terms of R^2^ compared to the variables entered later.

This new Table 1 shows R^2^ for each predictor computed using the *calc.relimp* function of the R package *relaimpo* (relative importance in linear models). The metric used is lmg, based on Lindeman, Merenda, and Gold (1980), which is a R^2^ partitioned by averaging sequential sums of squares over all orderings of the predictors, effectively correcting this situation. While the total R^2^ remains intact, the main difference of this new metric compare to the original is that for nearly all regional volume, age and sex have lower R^2^ (mean age: -7% (range: -12 to 0), sex: -4% (-10 to 0)) while eTIV and interactions have higher R^2^ (mean eTIV: 3% (-1 to 11), all interactions: 7% (0 to 15)) compared to the original results. Finally, there were very limited differences for scanner magnetic field strength and scanner manufacturer.

**Table 1:** Percentage of the variance explained (R^2^) by each predictor in models predicting subcortical regional volumes.

**Characteristic**	**Age**	**Age**^**2**^	**Age**^**3**^	**Sex**	**eTIV**	**eTIV**^**2**^	**eTIV**^**3**^	**MFS**	**GE / Siemens**	**Philips / Siemens**	**GE X MFS**	**Philips X MFS**	**eTIV X MFS**	**Age X Sex**	**eTIV X GE**	**eTIV X Philips**	**Total R**^**2**^	**Validation R**^**2**^
Accumbens L	18.0	0.2	-	1.6	1.4	0.1	-	3.3	1.0	0.6	1.2	2.7	-	8.4	-	-	38.5	34.2
Accumbens R	12.8	0.5	9.5	2.1	1.6	-	-	0.6	0.2	0.8	0.6	3.1	-	5.9	-	-	37.8	28.6
Amygdala L	4.9	1.2	5.1	7.0	8.8	0.2	-	7.2	0.3	0.2	0.6	0.8	-	2.4	1.4	1.5	41.4	39.0
Amygdala R	4.1	0.4	4.3	7.9	9.5	-	-	3.0	0.1	0.2	0.4	1.0	-	-	-	-	31.1	33.9
Brainstem	0.9	1.2	0.7	11.1	35.9	1.0	-	0.1	1.0	0.7	0.4	0.3	-	0.8	-	-	54.1	61.1
Caudate L	7.9	1.9	-	2.6	14.3	0.4	5.3	0.1	0.0	1.0	0.1	0.3	-	3.7	1.2	2.4	41.3	37.0
Caudate R	6.2	4.2	-	2.6	13.1	0.1	-	1.1	0.6	2.8	0.2	1.6	3.3	3.0	1.0	1.9	41.7	31.4
Hippocampus L	7.3	5.4	7.8	2.9	11.6	0.3	-	3.8	0.9	1.7	0.7	0.6	4.0	3.9	-	-	50.9	48.2
Hippocampus R	6.0	5.8	7.1	3.8	15.4	-	-	5.4	0.5	0.7	1.0	0.6	-	3.3	-	-	49.7	51.6
Pallidum L	6.1	1.9	4.2	5.2	13.5	0.3	-	2.9	0.4	0.2	0.2	0.9	-	4.1	-	-	40.0	37.8
Pallidum R	8.1	0.9	6.6	5.2	12.2	0.2	-	2.9	0.2	1.2	0.4	1.0	-	4.5	-	-	43.4	42.4
Putamen L	23.6	0.5	-	3.4	6.5	0.2	2.2	0.5	0.4	1.4	0.1	2.1	1.2	10.0	-	-	52.0	41.9
Putamen R	15.9	1.7	10.4	4.9	8.4	0.1	-	0.6	0.2	1.3	0.2	2.5	-	7.9	-	-	54.2	47.2
Thalamus L	10.6	2.3	7.5	4.6	18.1	0.8	-	2.4	0.2	1.0	0.5	1.0	7.0	5.3	-	-	61.5	57.3
Thalamus R	14.2	1.4	9.6	5.6	20.8	0.9	-	0.5	0.2	0.3	0.2	0.2	-	6.9	2.0	3.9	66.6	66.3
Ventral DC L	7.0	1.0	4.5	9.8	29.0	1.0	-	1.6	0.2	1.8	0.6	0.9	-	3.5	-	-	60.8	66.9
Ventral DC R	14.7	0.8	-	7.7	21.5	0.8	-	0.8	0.1	1.0	0.3	0.5	-	6.7	2.7	5.1	62.8	64.1
Ventricles	28.3	5.1	-	1.3	6.4	-	-	0.2	0.1	0.7	-	-	1.7	11.7	0.8	0.6	56.9	66.9
Lateral L^1^	27.6	3.9	-	1.1	6.8	-	-	-	0.1	0.6	-	-	-	11.7	0.8	0.8	53.4	61.7
Lateral R^1^	27.5	4.5	-	1.3	6.7	-	-	-	0.1	0.5	-	-	-	10.8	0.9	0.7	53.0	65.2
Inferior lateral L^1^	8.5	7.5	8.9	2.4	2.0	0.2	0.6	2.1	0.7	0.9	1.1	0.3	-	4.8	0.2	0.2	40.4	43.4
Inferior lateral R^1^	5.8	6.9	7.8	2.4	1.1	-	-	2.9	0.2	1.5	-	-	0.1	3.9	0.1	0.2	33.0	32.6
3rd^1^	19.5	4.1	15.3	3.0	6.3	0.1	-	0.1	0.3	0.2	0.1	0.4	2.1	8.4	-	-	59.9	64.1
4th	0.4	0.8	-	2.5	5.2	0.0	1.7	0.5	0.5	0.1	-	-	-	-	1.0	1.3	13.9	11.4
Corpus callosum	8.2	5.2	6.9	0.7	5.2	0.1	1.8	2.9	0.7	0.4	-	-	0.9	-	1.2	0.6	34.8	32.7
Subcortical GM	16.9	0.5	11.8	8.9	27.0	0.9	-	0.3	0.1	0.1	0.3	0.3	-	8.4	-	-	75.6	72.0

MFS: Magnetic field strength, eTIV: Estimated total intracranial volume. GM: gray matter.

We would like to apologize for any inconvenience caused.

