# Supplementary material for: FreeSurfer subcortical normative data
Source: Data Brief. 2016 Oct 14;9:732–6. doi: 10.1016/j.dib.2016.10.001 (PMC5094268; doi:10.1016/j.dib.2016.10.001)
Supplement: Supplementary file 1 — Supplementary material [file mmc3.docx]

**Acknowledgments**

Autism Brain Imaging Data Exchange (ABIDE): Primary support for the work by Adriana Di Martino was provided by the NIMH (K23MH087770) and the Leon Levy Foundation. Primary support for the work by Michael P. Milham and the INDI team was provided by gifts from Joseph P. Healy and the Stavros Niarchos Foundation to the Child Mind Institute, as well as by an NIMH award to MPM (R03MH096321). http://fcon_1000.projects.nitrc.org/indi/abide/

Alzheimer’s Disease Neuroimaging Initiative (ADNI): Funded by the ADNI (National Institutes of Health Grant U01 AG024904) and DOD ADNI (Department of Defense award number W81XWH-12-2-0012). ADNI is funded by the National Institute on Aging, the National Institute of Biomedical Imaging and Bioengineering, and through generous contributions from the following: AbbVie, Alzheimer’s Association; Alzheimer’s Drug Discovery Foundation; Araclon Biotech; BioClinica, Inc.; Biogen; Bristol-Myers Squibb Company; CereSpir, Inc.; Eisai Inc.; Elan Pharmaceuticals, Inc.; Eli Lilly and Company; EuroImmun; F. Hoffmann-La Roche Ltd and its affiliated company Genentech, Inc.; Fujirebio; GE Healthcare; IXICO Ltd.; Janssen Alzheimer Immunotherapy Research & Development, LLC.; Johnson & Johnson Pharmaceutical Research & Development LLC.; Lumosity; Lundbeck; Merck & Co., Inc.; Meso Scale Diagnostics, LLC.; NeuroRx Research; Neurotrack Technologies; Novartis Pharmaceuticals Corporation; Pfizer Inc.; Piramal Imaging; Servier; Takeda Pharmaceutical Company; and Transition Therapeutics. The Canadian Institutes of Health Research is providing funds to support ADNI clinical sites in Canada. Private sector contributions are facilitated by the Foundation for the National Institutes of Health (www.fnih.org). The grantee organization is the Northern California Institute for Research and Education, and the study is coordinated by the Alzheimer's Disease Cooperative Study at the University of California, San Diego. ADNI data are disseminated by the Laboratory for Neuro Imaging at the University of Southern California. http://adni.loni.usc.edu/

Australian Imaging Biomarkers and Lifestyle flagship study of ageing (AIBL): Part of the data used in this study was obtained from the Australian Imaging Biomarkers and Lifestyle flagship study of ageing (AIBL). See www.aibl.csiro.au for further details.

BMB - Berlin Mind and Brain (Margulies, Villringer). Zuo, X.N., et al. (2014). An open science resource for establishing reliability and reproducibility in functional connectomics. *Scientific data, 1*, 140049. doi: 10.1038/sdata.2014.49. http://fcon_1000.projects.nitrc.org/indi/CoRR/html/bmb_1.html

Cleveland Clinic (Cleveland CCF): Funded by the National Multiple Sclerosis Society. http://fcon_1000.projects.nitrc.org/indi/retro/ClevelandCCF.html

Center of Biomedical Research Excellence (COBRE): The imaging data and phenotypic information was collected and shared by the Mind Research Network and the University of New Mexico funded by a National Institute of Health COBRE: 1P20RR021938-01A2. http://fcon_1000.projects.nitrc.org/indi/retro/cobre.html

DS-108. Wager et al. (2008). Prefrontal-subcortical pathways mediating successful emotion regulation. Neuron, 59(6):1037-50. doi: 10.1016/j.neuron.2008.09.006. This data was obtained from the OpenfMRI database. NSF Grant OCI-1131441 (R. Poldrack, PI). Poldrack et al. (2013). Toward open sharing of task-based fMRI data: the OpenfMRI project. *Frontiers in neuroinformatics, 7*, 12. doi: 10.3389/fninf.2013.00012. https://openfmri.org/dataset/ds000108/

DS-170. Learning and memory: motor skill consolidation and intermanual transfer. This data was obtained from the OpenfMRI database. NSF Grant OCI-1131441 (R. Poldrack, PI). Poldrack et al. (2013). Toward open sharing of task-based fMRI data: the OpenfMRI project. *Frontiers in neuroinformatics, 7*, 12. doi: 10.3389/fninf.2013.00012. https://openfmri.org/dataset/ds000170/

Functional Biomedical Informatics Research Network (FBIRN): Provided by the Biomedical Informatics Research Network under the following support: U24-RR021992. http://www.birncommunity.org/resources/data/

FIND lab sample. Funded by the Dana Foundation; John Douglas French Alzheimer's Foundation; National Institutes of Health (AT005733, HD059205,HD057610, NS073498, NS058899).

http://fcon_1000.projects.nitrc.org/indi/retro/find_stanford.html

International Consortium for Brain Mapping (ICBM). http://www.loni.usc.edu/ICBM/

Information eXtraction from Images (IXI): Data collected as part of the project:

EPSRC GR/S21533/02 - http://www.brain-development.org/

F.M. Kirby Research Center neuroimaging reproducibility data (KIRBY-21). Landman, B.A. et al. “Multi-Parametric Neuroimaging Reproducibility: A 3T Resource Study”, NeuroImage. (2010) NIHMS/PMC:252138 doi:10.1016/j.neuroimage.2010.11.047 http://mri.kennedykrieger.org/databases.html

Minimal Interval Resonance Imaging in Alzheimer's Disease (MIRIAD): The MIRIAD investigators did not participate in analysis or writing of this report. The MIRIAD dataset is made available through the support of the UK Alzheimer's Society (RF116). The original data collection was funded through an unrestricted educational grant from GlaxoSmithKline (6GKC). http://miriad.drc.ion.ucl.ac.uk

Nathan Kline Institute Rockland (NKI-R) sample (phase 1) and (phase 2): Principal support for the enhanced NKI-RS project is provided by the [NIMH BRAINS R01MH094639-01](http://grants.nih.gov/grants/guide/rfa-files/RFA-MH-11-050.html). Funding for key personnel also provided in part by the New York State Office of Mental Health and Research Foundation for Mental Hygiene. Funding for the decompression and augmentation of administrative and phenotypic protocols provided by a grant from the [Child Mind Institute](http://childmind.org) (1FDN2012-1). Additional personnel support provided by the Center for the Developing Brain at the Child Mind Institute, as well as NIMH R01MH081218, R01MH083246, and R21MH084126. Project support also provided by the [NKI Center for Advanced Brain Imaging (CABI)](http://claymore.rfmh.org/), the [Brain Research Foundation](http://thebrf.org), the [Stavros Niarchos Foundation](http://www.snf.org/) and the NIH P50 MH086385-S1 (phase 1). http://fcon_1000.projects.nitrc.org/indi/pro/nki.html http://fcon_1000.projects.nitrc.org/indi/enhanced/

Open access series of imaging studies (OASIS): The OASIS project was funded by grants P50 AG05681, P01 AG03991, R01 AG021910, P50 MH071616, U24 RR021382, and R01 MH56584.http://www.oasis-brains.org/

Oulu FCON sample (Oulu). http://fcon_1000.projects.nitrc.org/fcpClassic/FcpTable.html

POWER: This database was supported by NIH R21NS061144 R01NS32979 R01HD057076 U54MH091657 K23DC006638 P50 MH71616 P60 DK020579-31 , McDonnell Foundation Collaborative Action Award, NSF IGERT DGE-0548890, Simon's Foundation Autism Research Initiative grant, Burroughs Wellcome Fund, Charles A. Dana Foundation, Brooks Family Fund, Tourette Syndrome Association, Barnes-Jewish Hospital Foundation, McDonnell Center for Systems Neuroscience, Alvin J. Siteman Cancer Center, American Hearing Research Foundation grant, Diabetes Research and Training Center at Washington University grant. http://fcon_1000.projects.nitrc.org/indi/retro/Power2012.html

Parkinson’s Progression Markers Initiative (PPMI): PPMI – a public-private partnership – is funded by the Michael J. Fox Foundation for Parkinson’s Research and funding partners, including Abbvie, Avid Radiopharmaceuticals, Biogen Idec, Bristol-Myers, Covance, GE Healthcare, Genentech, GlaxoSmithKline, Eli Lilly and Company, Lundbeck, Merck, Meso Scale Discovery, Pfizer, Piramal, Roche, and UCB. See http://www.ppmi-info.org for further details.

TRAIN-39: Data collected at the Biomedical Imaging Center at the Beckman Institute for Advanced Science and Technology at UIUC. Funded by the Office of Naval Research (ONR): N00014-07-1-0903. http://fcon_1000.projects.nitrc.org/indi/retro/Train-39.html

University of Wisconsin, Madison (Birn, Prabhakaran, Meyerand) CoRR sample (UWM). Zuo, X.N., et al. (2014). An open science resource for establishing reliability and reproducibility in functional connectomics. *Scientific data, 1*, 140049. doi: 10.1038/sdata.2014.49

http://fcon_1000.projects.nitrc.org/indi/CoRR/html/samples.html
