# Supplementary material for: FreeSurfer subcortical normative data
Source: Data Brief. 2016 Oct 14;9:732–6. doi: 10.1016/j.dib.2016.10.001 (PMC5094268; doi:10.1016/j.dib.2016.10.001)
Supplement: Supplementary file 2 — Supplementary material [file mmc1.docx]

**Conflict of interest**

O.P., A.M., and L.D. declare no competing financial interests. S.D. is officer and shareholder of True Positive Medical Devices Inc.
